# An effective method for culturing functional human corneal endothelial cells using a xenogeneic free culture medium

**DOI:** 10.1038/s41598-023-46590-2

**Published:** 2023-11-09

**Authors:** S. Alonso-Alonso, N. Vázquez, M. Chacón, N. Caballero-Sánchez, S. Del Olmo-Aguado, C. Suárez, B. Alfonso-Bartolozzi, L. Fernández-Vega-Cueto, L. Nagy, J. Merayo-Lloves, A. Meana

**Affiliations:** 1 Instituto Universitario Fernández-Vega, Fundación de Investigación Oftalmológica, Universidad de Oviedo, Avenida Doctores Fernández Vega, 33012 Oviedo, Asturias Spain; 2 Instituto de Investigación Sanitaria del Principado de Asturias (ISPA), Avenida del Hospital Universitario, 33011 Oviedo, Asturias Spain; 3 Doctoral School of Molecular Cell and Immunobiology. Faculty of Medicine, University of Debrecen, Nagyerdei Krt, Debrecen, 4032 Hungary; 4 Department of Biochemistry and Molecular Biology, Nuclear Receptor Research Laboratory, Faculty of Medicine, University of Debrecen, Nagyerdei Krt, Debrecen, 4032 Hungary; 5 Instituto Oftalmológico Fernández-Vega. Avenida Doctores Fernández-Vega, 33012 Oviedo, Asturias Spain; 6 Department of Medicine and Biological Chemistry, Johns Hopkins University School of Medicine, and Institute for Fundamental Biomedical Research, Johns Hopkins All Children’s Hospital, 6Th Ave S, St. Petersburg, FL 33701 USA; 7 Unidad de Ingeniería Tisular, Centro Comunitario Sangre y Tejidos de Asturias (CCST), Unidad 714 CIBERER, Calle Emilio Rodríguez Vigil, 33006 Oviedo, Asturias Spain

**Keywords:** Cell biology, Regenerative medicine

## Abstract

Endothelial dysfunction is a leading cause of corneal blindness in developed countries and the only available treatment is the endothelial transplantation. However, the limited availability of suitable donors remains a significant challenge, driving the exploration of alternative regenerative therapies. Advanced Therapy Medicinal Products show promise but must adhere to strict regulations that prohibit the use of animal-derived substances. This study investigates a novel culture methodology using Plasma Rich in Growth Factors (PRGF) as the only source of growth factors for primary cultures of human corneal endothelial cells (CECs). CECs were obtained from discarded corneas or endothelial rings and cultured in two different media: one supplemented with xenogeneic factors and other xenogeneic-free, using PRGF. Comprehensive characterization through immunofluorescence, morphological analyses, trans-endothelial electrical resistance measurements, RNA-seq, and qPCR was conducted on the two groups. Results demonstrate that CECs cultured in the xenogeneic-free medium exhibit comparable gene expression, morphology, and functionality to those cultured in the xenogeneic medium. Notably, PRGF-expanded CECs share 46.9% of the gene expression profile with native endothelium and express all studied endothelial markers. In conclusion, PRGF provides an effective source of xenogeneic-free growth factors for the culture of CECs from discarded corneal tissue. Further studies will be necessary to demonstrate the applicability of these cultures to cell therapies that make clinical translation possible.

## Introduction

The corneal endothelium is a neuroectoderm-derived tissue^1^ that is located on the posterior surface of the cornea forming a semipermeable monolayer of hexagonal and mitotically inactive cells resting over a collagen membrane called Descemet’s membrane^2^. Corneal Endothelial Cells (CECs) constitute a physiological tight intercellular barrier with the function of maintaining the corneal stroma in an adequate level of hydration^1^. When the function of these cells is compromised, corneal hydration increases causing corneal edema and thus modifying the alignment of the collagen fibers present in the stroma leading to a loss of transparency^3,4^.

The standard treatment of corneal endothelial dysfunction is corneal transplantation either as the whole cornea (penetrating keratoplasty) or as just the endothelial layer (lamellar keratoplasty)^5,6^. Endothelial cell failure constitutes the primary indication for corneal transplantation worldwide, being the indication for 55% of all keratoplasties performed in the US in 2018^7^. However, the worldwide supply of donor corneas is low, with only one cornea available per 70 patients. In addition, approximately one-third of donor corneas are discarded due to poor endothelial quality or the presence of infection^8^.

To overcome this shortage, researchers are exploring Advanced Therapy Medicinal Products (ATMP) as an alternative to conventional transplant procedures^9,10^. In this sense, the in vitro expansion of CECs using discarded cadaveric tissues seems an interesting approach for the development of tissue engineering^11–19^ or cell therapy based treatments^20–26^. Despite the promising result of these strategies, in vitro expansion of CECs is highly limited due to CECs being arrested in G1 phase^27^.

In the last few years, different culture media have been used for the expansion of CECs^28,29^*.* These methods often involve the use of fetal bovine serum (FBS), pituitary extract, and other xenogeneic products that are, in some cases, incompatible with the Good Manufacturing Practice (GMP) required for ATMP manufacture^30^.

The use of human blood derivatives such as platelet-rich plasma and platelet lysate products have demonstrated their efficacy for the in vitro expansion of CECs^31–34^. One of the most standardized human blood derivatives used is the Plasma Rich in Growth Factors (PRGF) (PRGF-Endoret^®^, BTI, Vitoria, Spain), a type of platelet-rich plasma consisting of plasma enriched in proteins and growth factors that stimulate and accelerate tissue regeneration^35,36^. Autologous PRGF has been approved for clinical use by the European Community and the U.S. Food and Drug Administration^37^, and it is generally employed as eye drops to treat ocular surface pathology^38–42^.

In this study, we investigated a novel method using PRGF as the unique source of growth factors in primary cultures of CECs. Through extensive characterization, we have demonstrated that the gene expression, morphology, and functionality of CECs cultured with PRGF culture medium are similar to CECs cultured with a standard FBS culture medium containing xenogeneic factors.

## Results

### Human tissues

CECs cultures were obtained using FBS (n = 31) or PRGF (n = 31) supplemented media. The age of the corneal donors and the corneal endothelial density, measured by specular microscopy, were calculated for each group (Table 1). No statistically significant differences were found between any group when comparing corneal endothelial density, determined prior to the surgery, or the age of the corneal donors.

**Table 1:** The age of the corneal donors and corneal endothelial density were determined prior to the hypothetical corneal graft by specular microscopy.

	PRGF (n = 31)		FBS (n = 31)	
Age (years)	Whole Corneal Endothelia (n = 13)	Endothelial Peripheral Rings (n = 18)	Whole Corneal Endothelia (n = 19)	Endothelial Peripheral Rings (n = 12)
	63 ± 3	63 ± 2	61 ± 2	60 ± 4
	63 ± 1		61 ± 2	
Corneal Endothelial Density (cell/mm^2^)	Whole Corneal Endothelia (n = 13)	Endothelial Peripheral Rings (n = 18)	Whole Corneal Endothelia (n = 19)	Endothelial Peripheral Rings (n = 12)
	2322 ± 167	2706 ± 46	2564 ± 124	2775 ± 79
	2560 ± 76		2658 ± 79	

Data are shown as the mean ± SEM. The normal distribution of values was assessed by the Shapiro–Wilk test. Significant differences among defined groups were checked using parametric tests (t-student or ANOVA test). PRGF: Plasma Rich in Growth Factors; FBS: Fetal Bovine Serum. SEM: Standard Error of Mean.

### Isolation and culture of CECs

**Figure 1:**
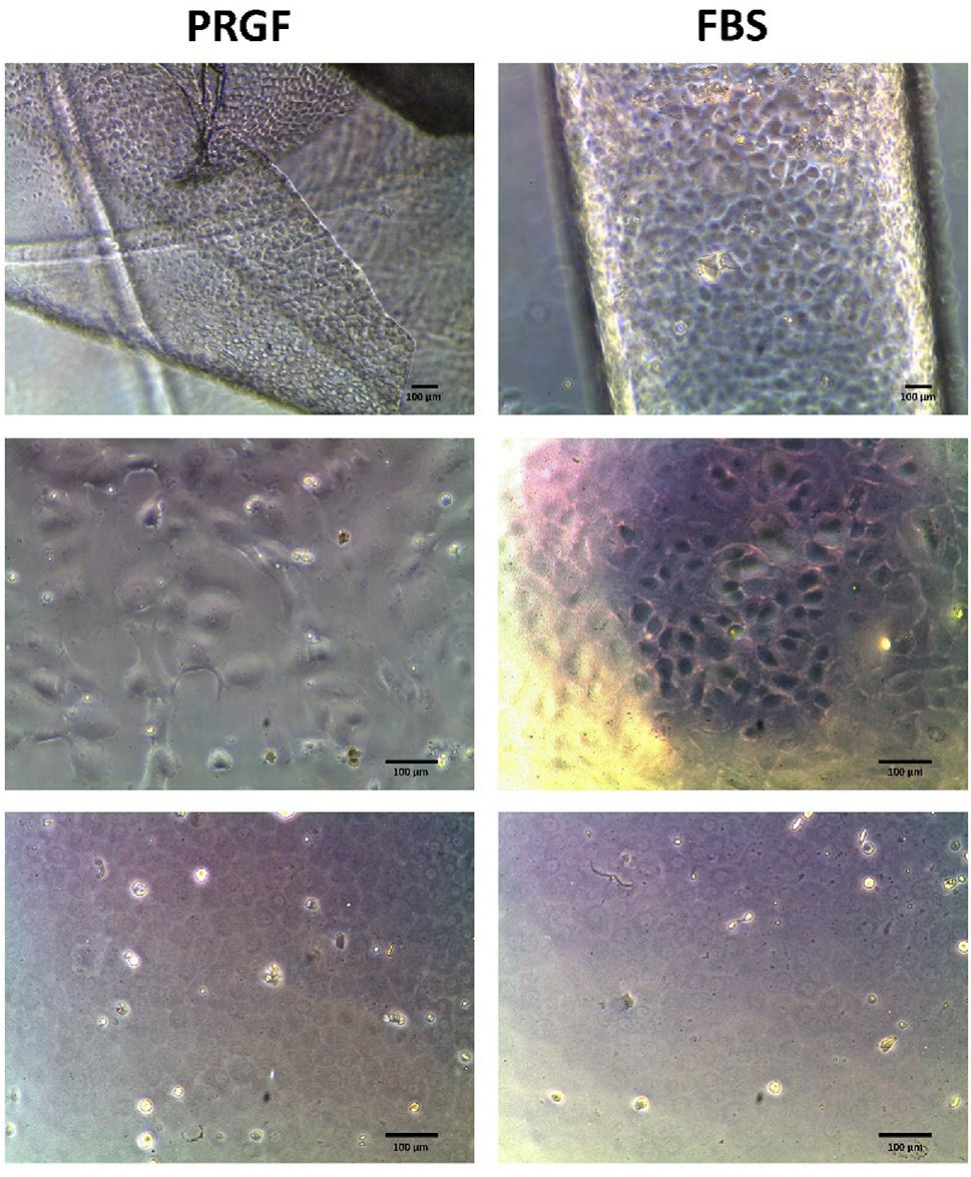
Phase-contrast microphotographs of CECs cultured in PRGF (left) or FBS (right) supplemented media. CECs in the native endothelium after the stabilization stage (first line), after 7 days of culture in P0 (second line), and displaying their typical hexagonal corneal endothelial morphology after 20–30 days of culture in P0 (third line). CECs: Corneal Endothelial Cells; PRGF: Plasma Rich in Growth Factors; FBS: Fetal Bovine Serum.

In all the cultures performed, CECs enhanced their typical hexagonal morphology in the corneal endothelium after the stabilization stage. After two days of the culture, CECs were attached and began to proliferate, showing cobblestone or polygonal morphology. By day 10–15, a monolayer of CECs had formed on all the culture plates. After 20–30 days of culture, confluent compact and hexagonal CECs cultures were obtained in all the tissues processed (Fig. 1).

### Immunocytochemistry, cellular density, and morphometric analysis

**Figure 2:**
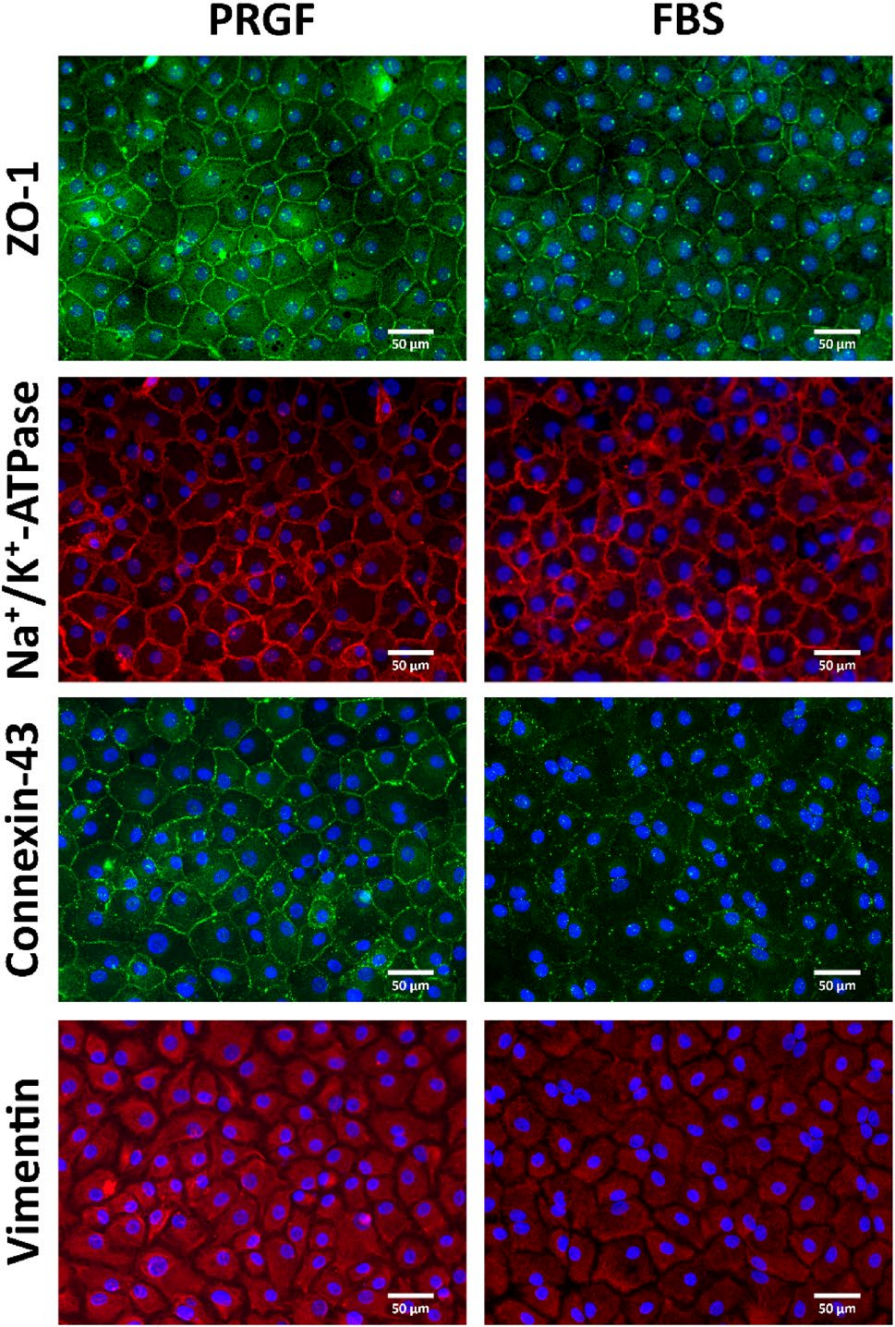
Immunofluorescence microphotographs of CECs cultured with PRGF or FBS-supplemented media in P0. CECs: Corneal Endothelial Cells; PRGF: Plasma Rich in Growth Factors; FBS: Fetal Bovine Serum. ZO-1: Zonula Occludens.

The immunocytochemistry analysis showed that CECs cultures in PRGF or FBS-supplemented media had a positive stain for the tight junction proteins Zonula occludens-1 (ZO-1) and connexin-43, functional protein Na^+^/K^+^-ATPase and structural protein vimentin (Fig. 2).

The cellular density and the morphometric analysis (Table 2) were determined by ZO-1 and Na^+^/K^+^-ATPase immunofluorescence and ImageJ analysis. CECs cultured in PRGF or FBS-supplemented media had a cellular density of 615.8 cell/mm^2^ ± 48.83 and 816.9 cell/mm^2^ ± 191.4, respectively, with a homogenous compact cellular morphology (area: 1249 ± 89.61 µm^2^ and 1386 ± 305.2 µm^2^, respectively) and hexagonal in shape as suggested by their cellular circularity (circularity index: 0.835 ± 0.01 and 0.801 ± 0.01, respectively). No statistically significant differences were found in the cellular density and area. However, the circularity index revealed a significantly higher value (p < 0.05) in the cultures supplemented with PRGF compared with ones supplemented with FBS.

**Table 2:** Cellular density (cell/mm^2^), area (µm^2^), and circularity index of CECs cultured in PRGF or FBS-supplemented media in P0.

	Cellular density (cell/mm^2^)	Area (µm^2^)	Circularity index
PRGF	615.8 ± 49	1249 ± 90	0.835 ± 0.01
FBS	816.9 ± 191	1386 ± 305	0.801 ± 0.01

Data are shown as the mean ± SEM. n = 6 for each group. The normal distribution of values was assessed by the Shapiro–Wilk test. Significant differences among defined groups were checked using parametric tests (t-student or ANOVA test). CECs: Corneal Endothelial Cells; PRGF: Plasma Rich in Growth Factors; FBS: Fetal Bovine Serum.

### Characterization of barrier function: trans-endothelial electrical resistance (TER)

CECs cultured in PRGF or FBS-supplemented media showed a TER value of 39.20 ± 4.09 Ωcm^2^ and 39.78 ± 2.83 Ωcm^2^ respectively. No statistically significant differences were found between groups.

### RNA isolation and sequencing (RNAseq)

To examine the capability of CECs cultured in PRGF or FBS-supplemented media to maintain a CECs-specific gene expression profile, bulk RNAseq was performed. Moreover, the gene expression of CECs cultured in PRGF or FBS-supplemented media was compared with native endothelium public data from Tokuda et al.^43^. Any gene across the genome with at least a count ≥ 1 was defined as expressed, while for the differential expression, the thresholds were a p-value < 0.05 and at least a fold change of 1.5.

23,744 out of 24,770 (91.86%) genes were found unchanged between CECs cultured in PRGF or FBS-supplemented media. On the other hand, 11,617 (46.90%) and 11,377 (45.93%) genes were found unchanged between CECs cultured in PRGF or FBS-supplemented media compared to native endothelium, respectively (Table 3).

**Table 3:** Unchanged and DEGs between the 3 groups of CECs: CECs cultured in PRGF or FBS supplemented medium in P0, and CECs in the native endothelium taken from public data^43^.

	PRGF vs FBS	Native Endothelium vs FBS	Native Endothelium vs PRGF
Unchanged	23,744	11,377	11,617
DEGs	1026	13,393	13,153
Upregulated	497	9177	9242
Downregulated	529	4216	3911

n = 4 for each group. DEGs: Differentially Expressed Genes; CECs: Corneal Endothelial Cells; PRGF: Plasma Rich in Growth Factors; FBS: Fetal Bovine Serum.

**Figure 3:**
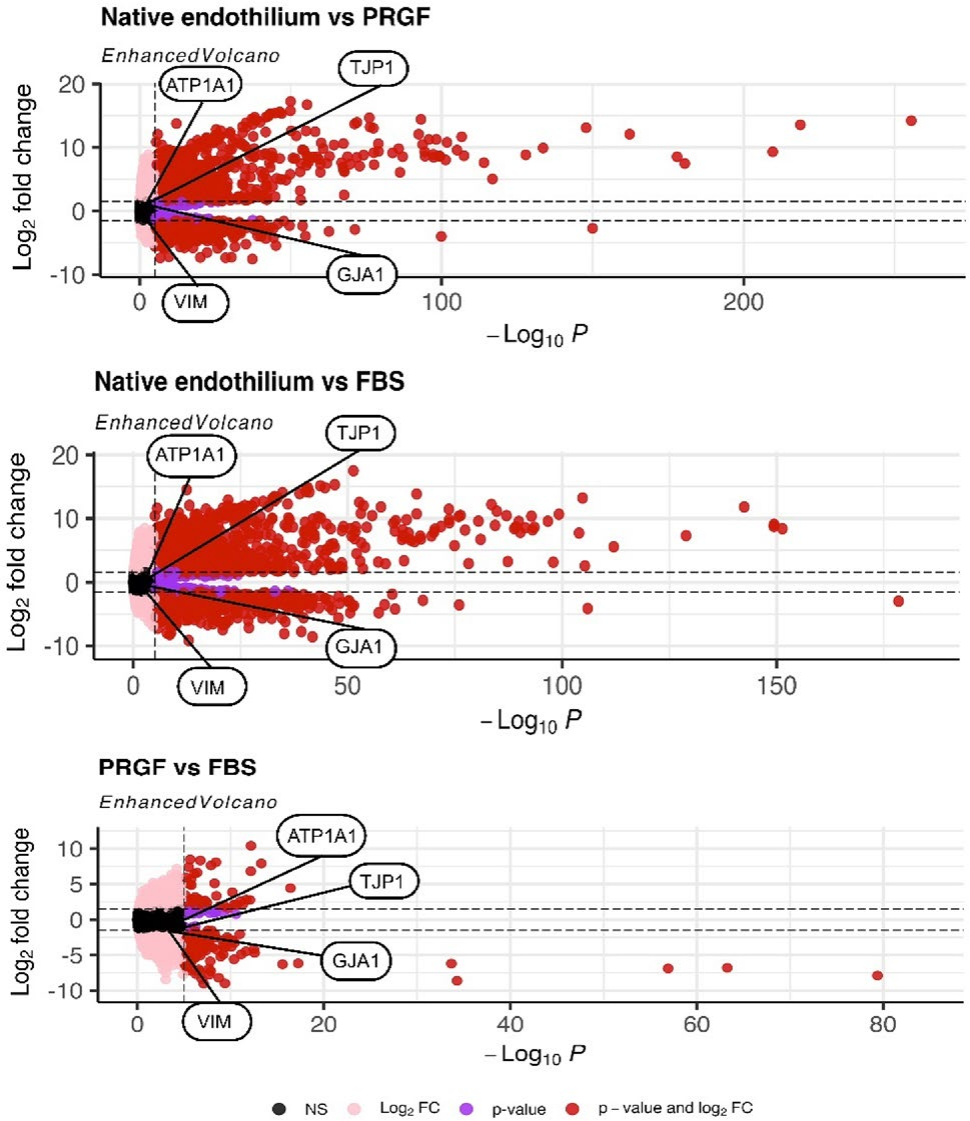
Volcano plot representing the DEGs between the three groups: CECs cultured in PRGF or FBS-supplemented media in P0 and CECs in the native endothelium public data^43^. The y-axis represents the log_2_ fold change of the gene expression, and the x-axis the p-value. The horizontal lines correspond to log_2_ foldchange <  − 1.5 and > 1.5, and the vertical line corresponds to -log_10_ (0.05), where 0.05 is the threshold for the p-value. The text boxes represent the position of the four functionality genes (*TJP1*: ZO-1; *ATP1A1*: Na^+^/K^+^ ATPase; *GJA1*: connexin-43 and *VIM*: vimentin). n = 4 for each group. DEGs: Differentially Expressed Genes; CECs: Corneal Endothelial Cells; PRGF: Plasma Rich in Growth Factors; FBS: Fetal Bovine Serum.

Moreover, a volcano plot (Fig. 3) representing the Differentially Expressed Genes (DEGs) between the groups is shown. No statistically significant differences in the four genes used as corneal endothelial markers (*TJP1*: ZO-1; *ATP1A1*: Na^+^/K^+^-ATPase; *GJA1*: connexin-43 and *VIM*: vimentin) were found.

**Figure 4:**
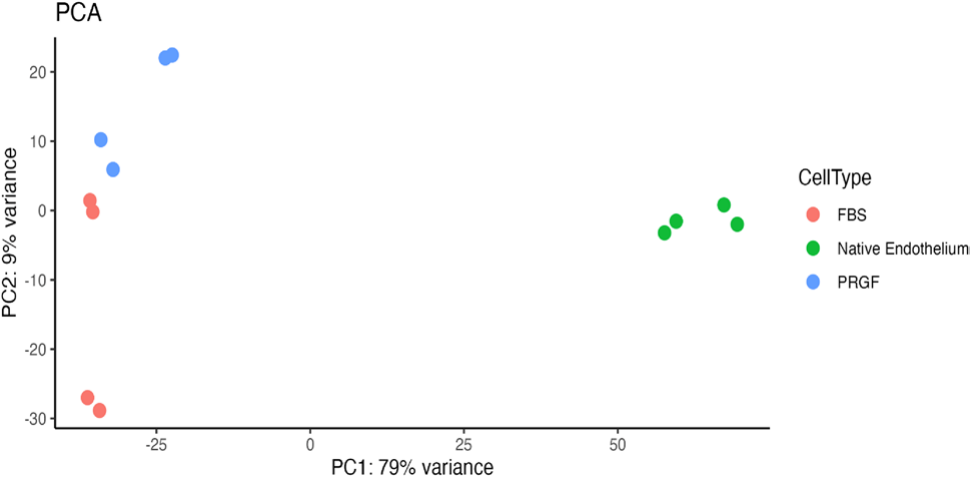
PCA results of CECs cultured in PRGF or FBS-supplemented media in P0 and native endothelium public data^43^. n = 4 for each group. PCA: Principal Component Analysis; CECs: Corneal Endothelial Cells; PRGF: Plasma Rich in Growth Factors; FBS: Fetal Bovine Serum.

Finally, Principal Component Analysis (PCA) was performed in RNA-seq data sets (Fig. 4). Gene expression data obtained by RNA-seq technology demonstrated the clustering of the samples into two distinct groups. One group was formed by CECs in the native endothelium, and the second was made up of CECs cultured in PRGF and FBS-supplemented media (PC1: 79%). The second principal component (PC2: 9%), represents the variance between CECs cultured in PRGF and FBS-supplemented media.

### RNA isolation and qPCR

**Figure 5:**
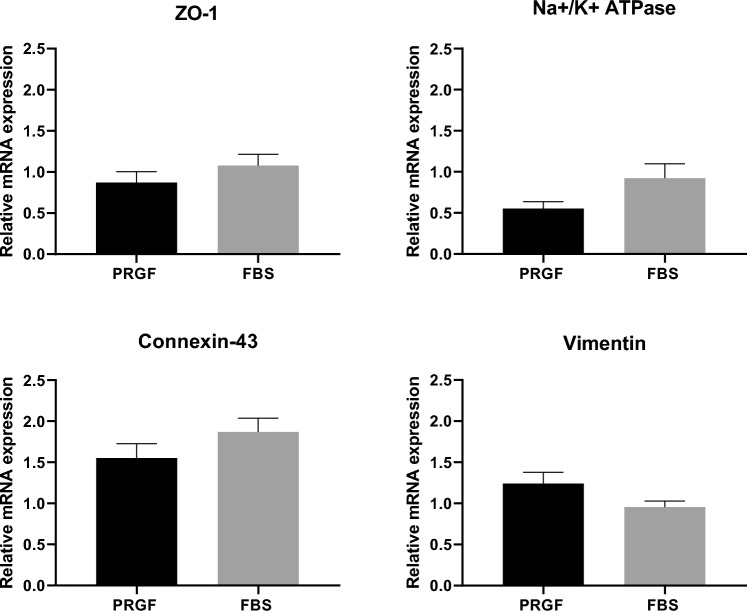
qPCR results of CECs cultured in PRGF or FBS-supplemented media in P0. Data are shown as the mean ± SEM. n = 7 for each group. The normal distribution of values was assessed by the Shapiro–Wilk test. Significant differences among defined groups were checked using parametric tests (t-student or ANOVA test). p-value 0.05. qPCR: quantitative Polymerase Chain Reaction. CECs: Corneal Endothelial Cells; PRGF: Plasma Rich in Growth Factors; FBS: Fetal Bovine Serum. SEM: Standard Error of Mean.

qPCR for the tight junction proteins ZO-1 and connexin-43, functional protein Na^+^/K^+^-ATPase and structural protein vimentin showed no statistically significant differences between CECs cultured in PRGF or FBS-supplemented media (Fig. 5).

## Discussion

Nowadays, the main way to restore vision in patients with severe endothelial damage is corneal transplantation from cadaveric donors. Surgical treatment has evolved in just over 100 years from complete corneal transplantation or penetrating keratoplasty, to selective transplantation of the damaged layer, first with Descemet-Stripping Automated Endothelial Keratoplasty (DSAEK) and later with DMEK, a less invasive technique that offers better visual results. Additionally, a novel cell therapy has gained approval for treating corneal edema in Japan, and it is under preparations in the US to commence clinical trials, and further plans for clinical development are in progress within the EU. However, both the traditional keratoplasties and the newer treatments rely on donated tissue.

The Spanish corneal transplant model is one of the most active in the world, with 4142 corneas transplanted in the year 2021 (87.4 corneal transplants per million inhabitants), according to Activity Report on Tissue Donation and Transplantation from Spanish National Transplant Organisation. Despite this, the aging of the population has led, in recent years, to a gap between the need for endothelial tissue and the number of donations and to an increase in the transplant waiting list. In this framework, the expansion of CECs for its use in Tissue Engineering or Cellular Therapy techniques is shown as an alternative which would reduce the waiting list for endothelial transplantation.

CECs in vitro expansion involves serious difficulties since CECs are arrested in the G1 phase when they are within their biological niche in the human eye^27^. This difficulty is evidenced by the many different culture techniques and culture media that have been used for CECs expansion^29,44^. Most of these media formulations incorporate xenogeneic elements, often utilizing FBS, thereby hindering the translation of these therapies into clinical application for various reasons. These difficulties encompass potential immune system responses to residual animal proteins^45^. To circumvent these limitations, the adoption of a xenogeneic-free culture method for CECs emerges as a solution, effectively avoiding some of these concerns.

In this study, CECs cultures (P0) were obtained using a standard culture medium containing xenogeneic factors and with a culture medium in which PRGF was used as the only supplement. CECs cultured in PRGF media showed similar characteristics in immunofluorescence, TER, qPCR, and RNAseq analyses to CECs cultured in standard culture medium.

CECs cultures were obtained from whole corneal endothelia from corneas discarded by Asturias Tissue Bank to be used in a transplant or from endothelial peripheral rings from corneas previously processed for DMEK surgery. After 30 days of culture, a monolayer of compact CECs displaying their typical hexagonal endothelial morphology was observed in both CECs cultures by phase-contrast microscopy and immunofluorescence. Immunohistochemical analysis revealed a positive stain for ZO-1 and connexin-43, tight junction-associated proteins responsible for establishing the endothelial barrier; Na^+^/K^+^ ATPase, an integral membrane protein mainly responsible for endothelial pump function; and vimentin, a structural protein useful to establish the baseline morphology. Immunostaining of ZO-1, connexin-43, and Na^+^/K^+^ ATPase clearly outlined the cell borders, which was concomitant with strong immunostaining of vimentin intermediate filaments.

ZO-1 and Na^+^/K^+^-ATPase immunofluorescence photographs were used to calculate cellular density and area with ImageJ software. The circularity index revealed a significantly higher value (p < 0.05) in the cultures supplemented with PRGF (0.835 ± 0.01) compared with ones supplemented with FBS (0.801 ± 0.01). Since hexagonal CECs will have a profile closer to 1 compared to long and spindly fibroblast-like cells^46^, CECs cultured in PRGF medium showed a more hexagonal morphology than CECs cultured in FBS, similar to that reported in previous studies, although with a more compact morphology^46^.

The leakiness of the endothelium is reflected in the very small TER of 15–25 Ω.cm^2^^47^. In this study, the resistance barrier, showed by ZO-1 and connexin-43 immunostaining, was confirmed by TER. CECs cultured in PRGF or FBS cultured media showed a small TER (39.20 ± 4.09 Ωcm^2^ and 39.78 ± 2.83 Ωcm^2^, respectively), although higher than previously reported in human CECs cultures^48^.

For a complete characterization of CECs, RNA-seq was also performed in four samples of each group. RNA-seq results for the four chosen markers showed similar expression levels of corneal endothelial markers in both CECs cultures as compared with native endothelium public data^43^, indicating that the culture does not affect the expression of the functional corneal endothelial markers selected. Despite the similar level of expression in the selected markers, a high number of DEGs between the data from cultured CECs and native endothelium were found. The differences between native endothelium and the cultures represent most of the variation between samples, as can be seen in the PCA. These results are in agreement with other RNA-seq studies where it was found that the culture of CECs affects gene expression in a progressive manner, increasing the differences along the number of cell passages^49,50^. qPCR was used to validate the expression level determined by RNAseq for the four genes studied. In accordance with RNAseq results, qPCR results showed non-significant differences between CECs cultured with PRGF or FBS media in the expression of the selected markers.

Several studies have demonstrated the efficacy of platelet-rich plasma and platelet lysate products for the in vitro expansion of CECs^31–34^. The novelty of our study lies in the fact that the PRGF is obtained with a protocol in which platelets are optimally concentrated, and immune cells are removed. Since white blood cells have often been suggested as a negative factor for tissue regeneration, PRGF could be a more suitable option for CECs culture than other platelet lysates^51^. Furthermore, since autologous PRGF has been approved for clinical use by the European Community and the U.S. Food and Drug Administration^37^, this culture methodology could facilitate the translation of any Tissue Engineering or Cellular Therapy technique into clinical practice.

Spanish legislation is very strict about the type of tissue that can be used in research, allowing only the use of discarded tissue for transplantation. Thus, in this study, sclerocorneal rings and corneas discarded due to not complying with the criteria for transplant were used. This fact, together with the age of the cadaveric donors (≈60 years), may have conditioned the limited CECs expansion obtained.

In considering the future translational potential of our methodology, it is essential to address the limited expansion observed in our study. Attempts to extend the expansion beyond the initial passages resulted in challenges, including the loss of endothelial morphology. The documented alterations^50^ in gene expression during cell passages highlight the complex dynamics associated with extended expansion. This fact has prompted a cautious approach, aiming to retain cellular characteristics while minimizing expansion.

Parekh et al. had discussed^52^ that during a DMEK surgery, a routine DMEK graft is 53.429 mm^2^; thus, assuming that in a standard DMEK graft, the CECs density is 2500 cells/mm^2^, resulting in a DMEK surgery with a total of 133,572 CECs being transplanted. These authors have also reported that it is possible to expand CECs from discarded tissues (peripheral endothelium or central endothelium) but using a methodology that includes xenogeneic factors ^53^.

In this study, we obtained the number of cells transplanted in a normal DMEK graft from one whole corneal endothelium unsuitable for clinic use or from two peripheral endothelial rings discarded from corneas previously processed for DMEK surgery. However, we use a methodology that is inexpensive and easy to transfer to clinical practice due to xenogeneic factors being avoided in the culture medium. Although we obtained a lower total number of CECs from peripheral endothelial rings (1 confluent P48 well) than from whole corneal endothelia (2 confluent P48 wells), CECs cultured in PRGF medium from peripheral endothelial rings or from whole corneal endothelia showed similar characteristics by immunofluorescence, TER, and qPCR analysis (data no showed).

In conclusion, CECs cultures can be obtained from discarded tissues for cell therapy or tissue engineering techniques using a methodology that is inexpensive and easy to transfer to clinical practice by avoiding the use of xenogeneic factors.

## Methods

### Human tissues

Human tissue was handled according to the Declaration of Helsinki. The research was approved by Ethics Committee of the Principado de Asturias (Comité de Ética de la Investigación del Principado de Asturias; Protocol Code: 2020.050). 51 corneas of 38 donors were obtained from the local Eye Bank (Centro Comunitario de Sangre y Tejidos, Oviedo, Asturias, Spain) after informed consent was obtained from the relatives, according to the Spanish transplant law RD Ley 9/2014. All corneas used in this study were discarded for clinical use due to not complying with the criteria to be transplanted: low corneal endothelial density (< 2000 cell/ mm^2^); corneal guttae; corneal striae with dead cells or anomalous morphology of CECs. Furthermore, sclerocorneal rings from corneas previously processed for Descemet Membrane Endothelial Keratoplasty (DMEK) surgery were also used.

All tissues were maintained at 4 ºC in Eusol-C storage medium (Alchimia, Ponte S. Nicolò, Padova, Italy) for fewer than 10 days before use. The mean age of corneal donors was 62 ± 1.78 years old, and the endothelial cell density of the corneas determined using a cell check specular endothelial microscope (Konan Medical, Irvine, CA, USA) was 2588 ± 64.93 cells/mm^2^.

### Preparation of PRGF

Blood from healthy volunteers (age range 25 – 45 years) was collected by venipuncture after informed consent was obtained from the subjects. They received an explanation of the nature and possible consequences of the study complying with the principles of the Declaration of Helsinki. The blood sample from each volunteer was processed according to the method described by Anitua et al.^54^. Briefly, 81 mL of human blood was collected into 9 mL tubes with 3.8% (wt/v) sodium citrate (Vacuette^®^, Greiner Bio-One, Madrid, Spain). Blood samples were centrifuged at 580 rcf for 8 min at room temperature, the plasma column was pipetted, avoiding the buffy coat, and incubated with 50 μL/mL of 10% calcium chloride (B. Braun Medical, Barcelona, Spain) at 37 ºC for 1 h, and finally, the released supernatants were collected by aspiration, filtered, aliquoted and stored at -20 ºC until use.

### Isolation and culture of CECs

CECs were isolated using a two-step “peel-and-digest” approach. Briefly, Descemet’s membrane, along with CECs, was carefully dissected from the corneal stroma under a dissecting stereomicroscope following the Schwalbe line, and then endothelial peripheral rings or whole corneal endothelia were maintained for 2–7 days at 37 °C to stabilize the cells before culture in either following culture medium:

FBS supplemented medium: optimem I (Life Technologies, Carlsbad, CA, USA) supplemented with 8%v/v FBS, 0.3 mM ascorbic acid 2-phosphate, 200 mg/L calcium chloride, 0.04% chondroitin sulfate, 10 U/mL penicillin, 10 µg/mL streptomycin, 20 ng/mL nerve growth factor (Merck, Darmstadt, Alemania), and 5 ng/mL epidermal growth factor (Austral Biologicals, San Ramon, CA, USA).

PRGF supplemented medium: optimem I supplemented with 10%v/v PRGF, 200 mg/L calcium chloride and 10 U/mL penicillin, and 10 µg/mL streptomycin.

Descemet’s membrane, along with CECs, was digested with TrypLE^®^ (Catalog number: 12605010; Thermo Fisher Scientific, Waltham, MA, USA) for 2 h at 37 °C. After that, TrypLE^®^ was neutralized with culture medium. The detached cells were centrifuged at 400 rcf for 10 min, the supernatant was removed, and the cells were seeded on one (endothelial peripheral rings) or two (whole corneal endothelia) wells of 48-well culture plates (1.10 cm^2^ area) previously treated with FNC coating mix^®^ (Athena Environmental Sciences, Baltimore, MD, USA). For characterization of barrier function, CECs were seeded on 1.12 cm^2^, 0.4 μm pore size Transwell^®^ inserts in a 12-well culture plate (Corning, Corning, NY, USA).

All incubation and cultures of CECs were carried out in a humidified incubator at 37 ºC with 5% CO_2,_ and the culture medium was changed every two–three days.

### Examination of cell cultures

Cellular growth was assessed by phase-contrast microscopy using a Leica DMIL LED phase-contrast microscope (Leica, Wetzlar, Hesse, Germany), and photos were taken with an attached EC3 camera (Leica). Once the cultures were confluent, they were used for the following studies.

### Immunocytochemistry, cellular density, and morphometric analysis

ZO-1 (1:50; Catalog number: 61–7300, Thermo Fisher Scientific), Na^+^/K^+^-ATPase a1 subunit (1:100; Catalog number: 05–369, Merck), connexin-43 (1:100; Catalog number: C6219, Merck) and vimentin (1:100; Catalog number: M0725, Agilent DAKO, Santa Clara, CA, USA) antibodies were used in order to check CECs phenotype. Briefly, confluent CECs cultures (n = 11 for each group) were fixed using ice-cold methanol (Merck) for 10 min, washed with PBS 1X solution twice for 5 min, and permeabilized in a PBS solution containing 0.3% Triton-X 100 (VWR, Radnor, PA, USA) for another 5 min (0.03% Triton-X 100 was used for ZO-1 antibody). Next, the samples were incubated with a primary antibody containing 10% normal goat serum (Catalog number: ab7481, Abcam, Cambridge, UK) at 4 °C overnight. Subsequently, the samples were incubated with corresponding secondary antibodies (1:500; Catalog number: A-11032 and A-11034, Thermo Fisher Scientific) for 2 h at room temperature. Between incubations, samples were washed 3 times with PBS for 10 min.

Immunolabeled cells were stained with 4′,6-diamidino-2-phenylindole (DAPI) (Thermo Fisher Scientific) to allow nuclei visualization. All the samples were examined using a Leica DM6000B fluorescence microscope, and 5 photos of random fields were captured using a Leica DFC310FX camera at 200 × magnification for morphological analysis.

ImageJ software v1.52t (NIH, Bethesda, MD, USA) was used to analyze cellular density and the morphology of CECs cultures in ZO-1 and Na^+^/K^+^ ATPase immunofluorescences (n = 6 for each group). The total number of cells was manually counted by two independent observers in the 5 photos captured, and morphometric data of the area, perimeter and circularity were manually measured by tracing cell borders in 2 random images. Cell circularity index was calculated as $\frac{4\uppi *\mathrm{area}}{{\mathrm{perimeter}}^{2}}$.The mean value and Standard Error of Mean (SEM) for each group were calculated.

### Characterization of barrier function: Trans-endothelial Electrical Resistance (TER)

A pair of Ag/AgCl probes and a Millicell-ERS2 volt-ohm meter (Merck) were used, according to the manufacturer guidelines, to evaluate the TER of confluent CECs cultures. TER value (Ωcm^2^) was calculated using the following equation:

$${\text{TER}} = \, ({\text{R}}_{{{\text{sample}}}} {-}{\text{ R}}_{{{\text{blank}}}} ) \, \times {\text{ effective area}}$$

$${\text{R}}_{{{\text{sample}}}} :{\text{ resistance value of the inserts with cultured CECs}}.$$

$${\text{R}}_{{{\text{blank}}}} :{\text{ resistance value of the inserts without cultured CECs}}.$$

$${\text{Effective area}}:{ 1}.{\text{12 cm}}^{{2}} .$$

All measurements (n = 9 for each group) were carried out in triplicate (three consecutive times in each culture), and the mean value and the SEM were calculated.

### RNA isolation and sequencing (RNAseq)

To obtain transcriptome data, mRNA sequencing analysis was performed on the Illumina sequencing platform. Total RNA from confluent cultures of CECs (n = 4 for each group) was extracted using the Pico Pure™ RNA isolation kit (Catalog number: KIT0202, Applied Biosystem, Foster City, CA, USA). Sample quality was checked on Agilent BioAnalyzer (Catalog number: G2939BA, Agilent) using Eukaryotic Total RNA Nano Kit (Catalog number: 5067–1511, Agilent) according to the Manufacturer’s protocol. The samples with RNA integrity number (RIN) value > 8 were accepted for the library preparation. The libraries were prepared from total RNA with NEBNext^®^ Ultra™ II RNA Library Prep for Illumina (New England BioLabs, Ipswich, MA, USA) according to the manufacturer’s protocol. Sequencing runs were executed on an Illumina NextSeq500 instrument (Illumina, San Diego, CA, USA) using single-end 75 cycles sequencing.

Native endothelium samples (DRR228774, DRR228775, DRR228778, DRR228780) FastQ files were downloaded from Sequence Read Archive (SRA). Reads were mapped to the human reference genome (GRCh38) using the default parameters of STAR v2.5.4. The quality control of the FastQ and BAM files was performed with MuliQC v1.13. Based on transcriptome alignment, as well as the genome alignment, featureCounts (Subread-v2.0.2) was used for obtaining the read counting to genes. Subsequent analysis was performed in R v4.2.2 and packages. Differential analysis was done with DEseq2 version 1.38.3.

Our data were also compared with public data from the study by Tokuda et al.^43^ Briefly in this study the data were obtained from CorneaGen corneas with a mean age of 61.14 years, All corneas had been stored at 4 °C in storage medium (Optisol-GS; Bausch & Lomb, Rochester, NY, USA) for less than 14 days before use for the experiments. Descemet's membrane, including the corneal endothelium, was removed from the donor corneas. The corneal endothelium was then lysed in 700 μL of QIAzol lysis reagent (Qiagen, Venlo, Netherlands). Total RNA was extracted from each corneal endothelium with an RNeasy Mini Kit (Qiagen) and RNA-Seq libraries for next-generation sequencing were generated with a SMARTer Stranded Total RNA-Seq Kit v2—Pico Input Mammalian (Takara Bio Inc., Shiga, Japan).

### mRNA isolation and quantitative PCR (qPCR)

Confluent CECs (n = 7 for each group) were digested with TrypLE^®^. Total RNA from CECs was extracted using the Pico Pure™ RNA isolation kit from Applied Biosystems™. The purity of the RNA was then checked by the A260/A280 and A260/A230 ratios. Next, total RNA was used for linear conversion of RNA to cDNA with High Capacity RNA-to-cDNA Master Mix (Catalog number: 4387406, Applied Biosystems) following the manufacturer’s instructions. Primers (Table 4) were customized using PrimerBLAST and synthesized by Merck. Gene expression was performed by relative quantification in a 7500 Real-Time PCR System (Applied Biosystems) using a Power SYBR Green PCR Master Mix (Catalog number: 4367659, Applied Biosystems) and the ∆∆Ct method. Each sample was analyzed in triplicate for each of the experiments, and the mean value and the SEM for each group were calculated. Data were analyzed using SDS 2.3 software (Applied Biosystems).

**Table 4:** Primers used in the qPCR analysis.

GENE	ID	FORWARD	REVERSE
*TJP1*	NM_001301025.3	5′-CAGCAACTTTCAGACCACCA-3′	5′-GTGCAGTTTCACTTGGCAGA-3′
*ATP1A1*	NM_000701.8	5′-TCCCAATTCACCTGTTGGGC-3′	5′-TGCACCACCACGATACTGAC-3′
*CX43*	NM_000165.5	5′-AGGAGTTCAATCACTTGGCGT-3′	5′-TACTGACAGCCACACCTTCC-3′
*VIM*	NM_003380.5	5′-CTCCCTGAACCTGAGGGAAACT-3′	5′-AGGTCACGTGATGCTGAGAAG-3′
*ACTB*	NM_001101.5	5′-ATTCCAAATATGAGATGCGTTGTT-3′	5′-GTGGACTTGGGAGAGGACTG-3′

### Statistical analysis

Statistical analyses were performed using GraphPad Prism v8.0.1 (GraphPad, San Diego, CA, USA). The normal distribution of tested values was assessed by the Shapiro–Wilk test. Significant differences among defined groups were checked using parametric tests (t-student or ANOVA test). Differential levels with p < 0.05 were statistically significant, and p < 0.01 was statistically very significant. Data are expressed as the mean ± SEM.

## Data Availability

The RNA-seq data presented in this article have been deposited in GEO under accession number GSE228460.
